# HfAlO_x_/Al_2_O_3_ Bilayer Dielectrics for a Field Effect Transistor on a Hydrogen-Terminated Diamond

**DOI:** 10.3390/ma15020446

**Published:** 2022-01-07

**Authors:** Minghui Zhang, Fang Lin, Wei Wang, Feng Wen, Genqiang Chen, Shi He, Yanfeng Wang, Shuwei Fan, Renan Bu, Hongxing Wang

**Affiliations:** 1 Key Lab for Physical Electronics and Devices, Ministry of Education, Xi’an Jiaotong University, Xi’an 710049, China; zhangminghuicc@mail.xjtu.edu.cn (M.Z.); leaf-lin@mail.xjtu.edu.cn (F.L.); fengwen@mail.xjtu.edu.cn (F.W.); genqiangchen@stu.xjtu.edu.cn (G.C.); thekeith@stu.xjtu.edu.cn (S.H.); yanfengwang@stu.xjtu.edu.cn (Y.W.); shwfan@mail.xjtu.edu.cn (S.F.); bura@xjtu.edu.cn (R.B.); 2 Institute of Wide Band Gap Semiconductors, School of Electronics and Information Engineering, Xi’an Jiaotong University, Xi’an 710049, China

**Keywords:** hydrogen-terminated diamond, field effect transistor, HfAlO_x_

## Abstract

In this work, a hydrogen-terminated (H-terminated) diamond field effect transistor (FET) with HfAlO_x_/Al_2_O_3_ bilayer dielectrics is fabricated and characterized. The HfAlO_x_/Al_2_O_3_ bilayer dielectrics are deposited by the atomic layer deposition (ALD) technique, which can protect the H-terminated diamond two-dimensional hole gas (2DHG) channel. The device demonstrates normally-on characteristics, whose threshold voltage (V_TH_) is 8.3 V. The maximum drain source current density (I_DSmax_), transconductance (G_m_), capacitance (C_OX_) and carrier density (ρ) are −6.3 mA/mm, 0.73 mS/mm, 0.22 μF/cm^2^ and 1.53 × 10^13^ cm^−2^, respectively.

## 1. Introduction

The diamond is considered as an ultimate semiconductor with ultrawide bandgap of 5.47 eV, extremely high breakdown field of 10 MV/cm, highest thermal conductivity of 22 W/cm·K, high carrier mobility (electrons of 4500 cm^2^/V·s, holes of 3800 cm^2^/V·s) and large carrier saturation velocity (electrons of 1.5–2.7 × 10^7^ cm/s, holes of 0.85–1.2 × 10^7^ cm/s) [1,2,3,4]. Since the dopants cannot be activated at room temperature with high activation energy (boron of 370 meV and phosphorous of 650 meV) [5], the application of diamonds has been greatly hindered. In this case, δ-doping comes into being. However, this technique requires the precise control of the doping thickness, and the carrier mobility is not ideal [5]. Fortunately, a hydrogen-terminated (H-terminated) diamond with two-dimensional hole gas (2DHG) channel provides a new solution to overcome these problems, which demonstrates a high carrier density of 10^13^ cm^−2^ and large carrier mobility of 50–200 cm^2^/V·s [3,6]. To date, as a promising structure of diamond-based electronic devices, the H-terminated diamond field effect transistor (FET) has aroused the great interest of researchers [7,8,9,10,11,12,13,14,15,16,17,18,19,20,21].

Since a H-terminated diamond is thermally and chemically instable, it is necessary to stabilize the hole carriers for a H-terminated diamond FET with a dielectric layer [8]. Furthermore, the dielectric material with high dielectric constant can control large charge responses at a small bias effectively [14]. To date, many high dielectric constant materials have been employed for the fabrication of a H-terminated diamond FET [10,15]. However, there are few reports on using HfAlO_X_ as dielectric with a high dielectric constant, high crystallization temperature and large band gap (5.8–6.2 eV). [22].

In this work, we study a H-terminated diamond FET with HfAlO_x_/Al_2_O_3_ bilayer dielectrics, and its electrical properties were evaluated by semiconductor analyzer.

## 2. Materials and Methods

**Figure 1:**
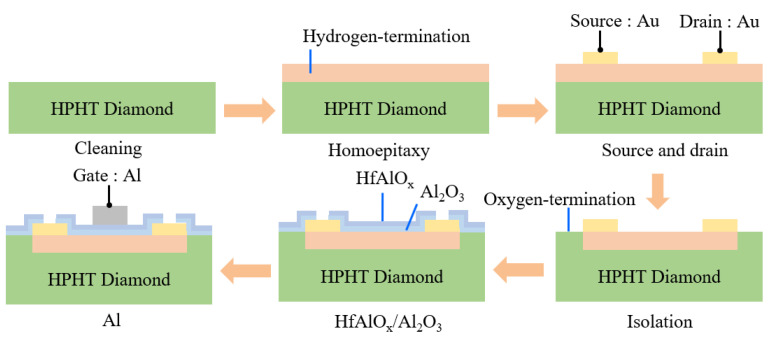
Fabrication process of the H-terminated diamond FET with HfAlO_x_/Al_2_O_3_ bilayer dielectrics.

**Figure 2:**
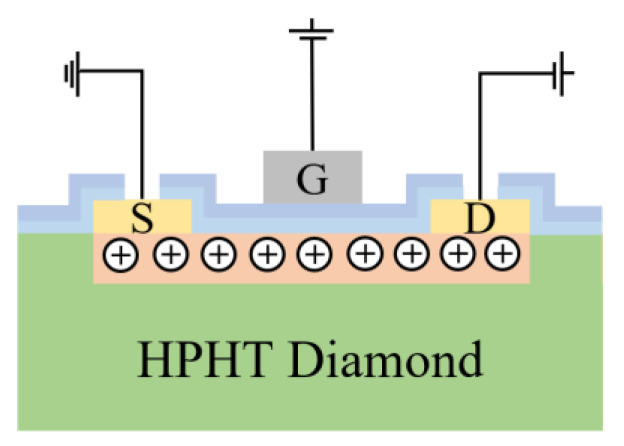
Schematic diagram of the H-terminated diamond FET with HfAlO_x_/Al_2_O_3_ bilayer dielectrics.

The fabrication process of the H-terminated diamond FET with HfAlO_x_/Al_2_O_3_ bilayer dielectrics is displayed in Figure 1. A high temperature and high pressure (HPHT) single crystal diamond substrate was cleaned by various solutions before growth [9]. Then, a 200 nm homoepitaxy layer was grown on the substrate with the dimensions of 3 × 3 × 0.5 mm^3^ by the microwave plasma chemical vapor deposition (MPCVD) technique. The growth conditions were declared in our previous work [9]. Afterwards, 150 nm Au electrodes with 20 μm source drain gap (L_SD_) were realized by photolithography, electron beam evaporation (EB) and the lift-off technique. Next, isolation was carried out with 20 min UV/ozone treatment. After that, a 4 nm Al_2_O_3_ film was deposited to protect the H-terminated channel, and a 30 nm HfAlO_x_ film was deposited by the ALD technique sequentially. The atomic percentage of HfAlO_x_ is Hf:Al:O = 2:23:75, evaluated by the energy dispersive X-ray spectroscopy (EDS) technique. Finally, 150 nm Al gate electrode was deposited on the gate region with 4 μm gate length (L_G_) and 100 μm gate width (W_G_). The electrical properties of this device were characterized by Agilent B1505A. Figure 2 demonstrates the schematic diagram of the H-terminated diamond FET with HfAlO_x_/Al_2_O_3_ bilayer dielectrics. The electrical contacts for the source, drain and gate electrodes are exhibited, and the hole carriers of the channel are illustrated.

## 3. Results and Discussion

**Figure 3:**
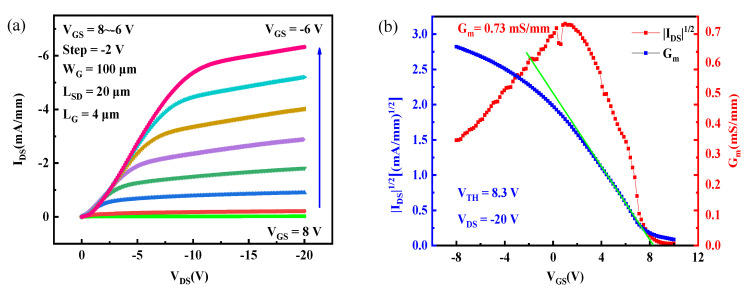
Characteristics of the H-terminated diamond FET with HfAlO_x_/Al_2_O_3_ bilayer dielectrics: (**a**) output and (**b**) transfer.

Figure 3a demonstrates the drain source current density (I_DS_) versus drain source voltage (V_DS_) at different gate voltages (V_GS_) of the H-terminated diamond FET with HfAlO_x_/Al_2_O_3_ bilayer dielectrics. The gate length (L_G_), gate width (W_G_) and L_SD_ for the device are 4 μm, 100 μm and 20 μm, respectively. The V_GS_ varies from 8 to −6 V in a step of −2 V. The absolute value of I_DS_ (|I_DS_|) increases as the absolute value of the V_GS_ (|V_GS_|) increases, indicating the existence of a p-type channel. The maximum I_DS_ (I_DSmax_) is −6.3 mA/mm obtained at a V_GS_ of −6 V and a V_DS_ of −20 V. The I_DSmax_ is relatively large compared with our previous work [13,23], and the reason may be attributed to the undamaged 2DHG conduction channel protected by Al_2_O_3_.

In Figure 3b, the transfer characteristic of the H-terminated diamond FET with HfAlO_x_/Al_2_O_3_ bilayer dielectrics is presented. The threshold voltage (V_TH_) is extrapolated to be 8.3 V at a V_DS_ of −20 V based on the relationship between |I_DS_|^1/2^ and V_GS_, demonstrating normally-on characteristics [14]. The maximum transconductance (G_m_) is 0.73 mS/mm.

**Figure 4:**
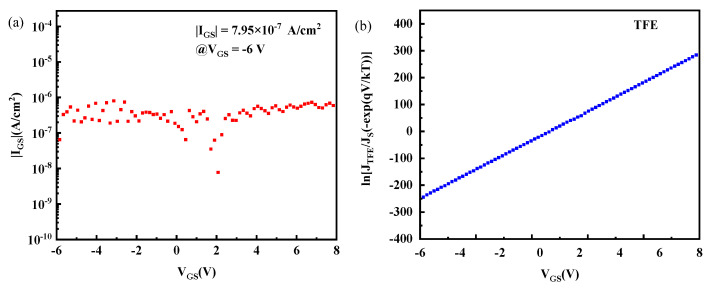
I_GS_ characteristics of the H-terminated diamond FET with HfAlO_x_/Al_2_O_3_ bilayer dielectrics: (**a**) |I_GS_| and (**b**) TFE.

The leakage current density (I_GS_) in the log coordinate of the H-terminated diamond FET with HfAlO_x_/Al_2_O_3_ bilayer dielectrics is shown in Figure 4a. The V_GS_ changes from −6 to 8 V, and the absolute value of I_GS_ (|I_GS_|) is 7.95 × 10^−7^ A/cm^2^ at a V_GS_ of −6 V, demonstrating a low |I_GS_|. Table 1 demonstrates the |I_GS_| comparison with the reported H-terminated FETs. The |I_GS_| for the MoO_3_, LiF/Al_2_O_3_, Ta_2_O_5_/Al_2_O_3_ and ZrO_2_/Al_2_O_3_ H-terminated diamond FET are 3.33 × 10^−4^ A/cm^2^, 1 × 10^−6^ A/cm^2^, 7.6 × 10^−4^ A/cm^2^ and 4.8 × 10^−5^ A/cm^2^, respectively [21,24,25,26]. Their values are larger than those of the HfAlO_x_/Al_2_O_3_ FET. As shown in Figure 4b, the relationship between I_GS_ and V_GS_ can be described by the thermionic field emission (TFE) model (1) [9]:

(1) $$J_{\mathrm{TFE}}=J_{S}\exp\left(V/P\right)\left[1-\exp\left(-\mathrm{eV}/\mathrm{kT}\right)\right]$$

where J_TFE_ means the I_GS_ caused by TFE model; J_S_ represents the saturation current; and P is a parameter associated with the carrier tunneling probability and temperature [9]. In Figure 4b, the lnJ_TFE_/J_S_(−exp(qV/kT)) and V_GS_ exhibit a linear relationship under the TFE model.

**Figure 5:**
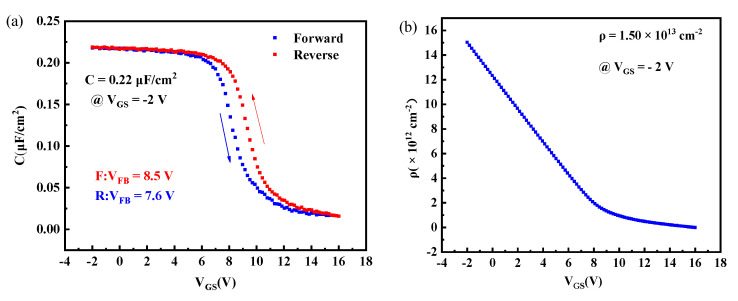
Characteristics of the H-terminated diamond FET with HfAlO_x_/Al_2_O_3_ bilayer dielectrics measured at 1 MHz: (**a**) C-V and (**b**) ρ.

Figure 5a displays the capacitance-voltage (C-V) curve measured at 1 MHz of the H-terminated diamond FET with HfAlO_x_/Al_2_O_3_ bilayer dielectrics. Evident accumulation and depletion regions can be observed. The maximum capacitance (C_OX_) is 0.22 μF/cm^2^ at V_GS_ of −2 V. Based on the method d^2^C/d^2^V_GS_ = 0, the flat band voltage (V_FB_) is determined to be 8.5 V and 7.6 V in the forward and reverse directions, respectively [18]. The trapped charge density is evaluated to be 1.24 × 10^12^ cm^−2^ based on the hysteresis voltage of 0.9 V [8]. Additionally, the CV curve shifts to the positive direction, indicating the presence of fixed negative charge in the dielectric layer. This increases the hole carriers in the 2DHG channel, thus resulting in a normally-on operation. The fixed negative charge density is deduced to be 1.25 × 10^13^ cm^−2^ on the basis of C_OX_, V_FB_, and the work function difference between Al and the H-terminated diamond [8]. In addition, the dielectric constant of HfAlO_x_/Al_2_O_3_ is calculated to be 8.45, suggesting that the quality of HfAlO_x_/Al_2_O_3_ needs to be further improved. As demonstrated in Figure 5b, the carrier density (ρ) is 1.50 × 10^13^ cm^−2^ at the V_GS_ of −2 V evaluated based on $\int^{\;}CdV_{\mathrm{GS}}$ [18]. The ρ is large, and this can be attributed to the high quality of the H-terminated diamond [8,21].

## 4. Conclusions

In summary, the electrical properties of H-terminated diamond FET with HfAlO_x_/Al_2_O_3_ bilayer dielectrics were investigated. The output characteristics demonstrate an evident p-type channel, and the I_DSmax_ is −6.3 mA/mm obtained at V_GS_ of −6 V. The transfer characteristics exhibits the V_TH_ of 8.3 V, indicating normally-on characteristics. The |I_GS_| is 7.95 × 10^−7^ A/cm^2^ at a V_GS_ of −6 V, demonstrating a low |I_GS_|. In addition, the C_OX_ is 0.22 μF/cm^2^ based on the C-V curve. Additionally, the ρ is 1.50 × 10^13^ cm^−2^ at a V_GS_ of −2 V. The results are meaningful for the research of a H-terminated diamond FET, and the electrical performance of HfAlO_x_/Al_2_O_3_ FET will be further improved by optimizing the fabrication process in our future work.

## Figures and Tables

**Table 1:** The |I_GS_| comparison between this work and the reported H-terminated diamond FETs.

Gate Materials	MoO_3_	LiF/Al_2_O_3_	Ta_2_O_5_/Al_2_O_3_	ZrO_2_/Al_2_O_3_	HfAlO_x_/Al_2_O_3_
|I_GS_| (A/cm^2^)	3.33 × 10^−4^	1 × 10^−6^	7.6 × 10^−4^	4.8 × 10^−5^	7.95 × 10^−7^
Ref.	[21]	[24]	[25]	[26]	This work

## Data Availability

Data are contained within the article.
